# Spontaneous bladder rupture after non-traumatic vaginal delivery: a rare case report

**DOI:** 10.11604/pamj.2023.44.22.33702

**Published:** 2023-01-12

**Authors:** Yasmine Ellouze, Maroua Abdelmoula, Sahar Elleuch, Anouar Jarraya, Khaled Trigui, Fatma Chaker, Smaoui Walid, Kolsi Kamel

**Affiliations:** 1 Department of Anesthesiology and Intensive Care, Hedi Chaker University Hospital, Sfax, Tunisia,; 2 Department of Gynecology and Obstetrics, Hedi Chaker University Hospital, Sfax, Tunisia,; 3 Department of Urology, Habib Bourguiba University Hospital, Sfax, Tunisia

**Keywords:** Bladder rupture, vaginal delivery, laparotomy, case report

## Abstract

Spontaneous bladder rupture (SBR) is a rare condition and often missed diagnosis, especially after a non traumatic vaginal delivery. A 32-year-old para 3 woman, consulted for abdominal pain and anuria two days after instrumental vaginal delivery with forceps for foetal distress in second sate of labour. Blood tests were suggestive of an acute renal failure. An abdominocentesis revealed a clear fluid looking like ascites. The ultrasound and computed tomography (CT) scan showed a large abdominal effusion. An exploratory laparoscopy revealed a bladder perforation which was sutured after laparotomy. SRB is extremely rare after a non traumatic vaginal delivery. It is associated with significant morbidity and mortality. Symptoms are mostly non-specific. It is suspected when post partum abdominal pain is associated with an effusion and renal failure signs. If suspected, the uroscanner remains the gold standard for diagnostic. Laparotomy is the standard surgical approach in this condition. Abdominal pain with elevated serum creatinine should be suspicious of SBR in post-partum.

## Introduction

Spontaneous rupture of the bladder (SRB) is a rupture that occurs in a healthy bladder without traumatic history [1,2]. Its usual clinical manifestations are abdominal pain and effusion [2]. During childbirth, SRB happens rarely (13 cases were reported in the literature since 1990 to 2020) [2]. However, it causes high maternal morbidity and mortality [1,2]. That´s why it is a diagnostic and therapeutic emergency. Its treatment consists on a surgical repair. We report the case of a patient who had SRB following an instrumental vaginal delivery with forceps.

## Patient and observation

**Patient information and clinical history:** we present a case report of a woman who consulted a regional hospital for abdominal pain and anuria two days after vaginal forceps delivery. She was 32 years old and had no significant medical or surgical history. She had had two previous spontaneous uncomplicated vaginal deliveries. The index delivery was her third spontaneous instrumental vaginal delivery following an uneventful pregnancy.

**Clinical findings:** the vaginal delivery was assisted by abdominal compression maneuver and then forceps for fetal distress in second stage of labour. No complication was recorded. The delivery was performed by an obstetrician in a peripheral regional hospital. Two days after, she reconsulted the same hospital for abdominal pain. The physical examination revealed generalized abdominal tenderness and distension associated with dullness at the flanks. Blood tests revealed features suggestive of acute renal failure (creatinine = 868 µmol/l/urea = 40 mmol/l) (normal reference ranges of creatinine (39-89 µmol/l) and of urea (2.6-7.2 mmol/l)). Laboratory tests revealed also elevated white blood cells to 18,000 elements/mm^3^, elevated C-reactive protein (CRP) to 455 mg/l and elevated fibrinogen to 10 g/l (normal white blood cells <10000 elements/mm^3^, normal CRP <6 mg/l and normal reference ranges of fibrinogen (2-5 g/l)). An abdominocentesis revealed clear fluid looking like ascites. The patient had a session of dialysis and was then transferred to our university-hospital. On examination, at our facility, her overall clinical condition was good, with a normal blood pressure (118/78 mmHg) and tachycardia (pulse rate was 120 beats per minute).

**Diagnostic assessment:** bacteriological investigation was done (blood culture, endocervical swab, urine culture and COVID-19 polymerase chain reaction (PCR)). Empirical broad-spectrum antibiotic therapy was administered intravenously without waiting for the bacteriological results (imipinem 1g/8hours, ciprofloxacin 200mg/12 hours and metronidazole 0.5g/8 hours). The chest X-ray showed bilateral basal alveolar opacities. The ultrasound and CT scan showed a large abdominal effusion. Therefore, a laparoscopic exploration was decided.

**Figure 1:**
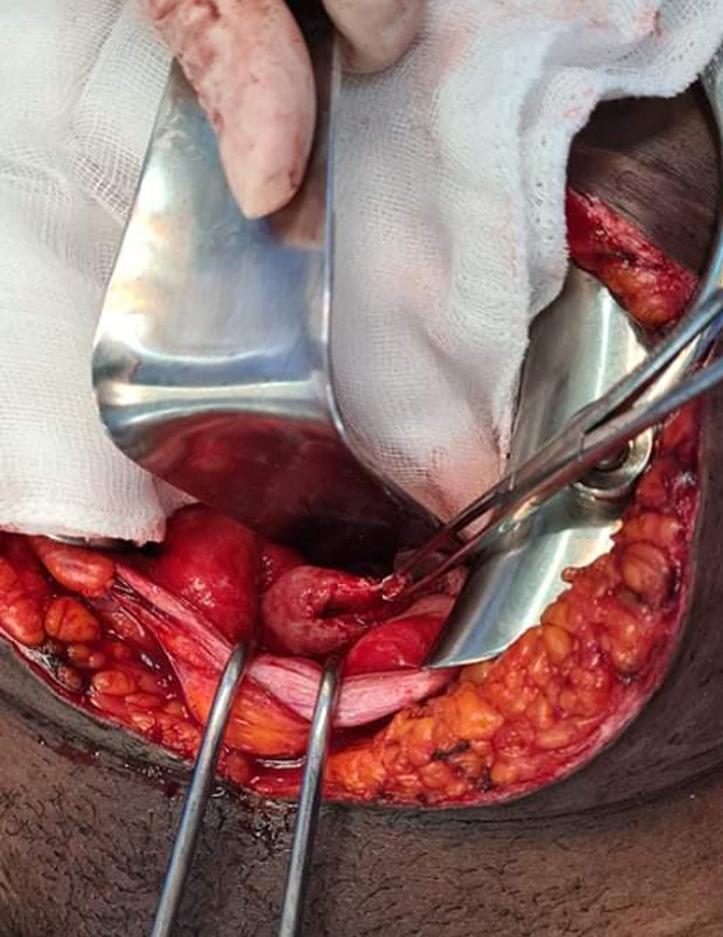
bladder rupture

**Therapeutic intervention:** during this procedure, a bladder perforation was strongly suspected when air was observed in the urine bag after coelioscopic insufflation. Because of technical difficulties due to inflammatory adhesions, the laparoscopy was converted to laparotomy. About 1.5 liters of ascitic fluid was drained. Exploration revealed a 2 cm perforation of the bladder´s dome with jagged edges partially clogged by a grelic handle (Figure 1). The bladder perforation was sutured by a urologist. Post operatively, the antibiotic therapy was changed to 1g of cefotaxim three times daily for 10 days, based on the endocervical swab culture result which revealed *Klebsiella pneumonia* sensitive to cefotaxim. All other bacteriological culture results were negative.

**Follow-up and outcomes:** the postoperative course was good. The bladder catheter was kept for ten days.

**Informed consent:** written informed consent was obtained from the patient for publication of this case report and accompanying images.

## Discussion

During post-partum period, many pathologies can be revealed by an abdominal pain and ascites. The etiological diagnosis can thus be difficult. One of the possible causes is SRB. It is an extremely rare (1: 126 000) [1] but a serious condition causing high morbidity and mortality (47%) [2]. The few cases reported in the literature were by Stabile *et al*. in a recently published review of 13 cases [2].

The reported median onset of SRB was three days [2]. Usually, SRB occurs following a bladder damage (from malignant diseases, acute or chronic urinary distension, obstructive pathology, chronic infection, necrotizing or post-radiation cystitis) or bladder trauma or a combination of the two [3,4]. The occurrence of peri-partum SRB has been reported in the literature and generally was associated with an obstructed labor or a scarred uterus [5].

Many of the risk factors have been deduced from the reported cases: a history of a caesarean section, instrumental vaginal delivery and bladder distension and compression during engagement of the foetal head in labour. If the latter is prolonged, necrosis of the bladder dome may occur. Bladder distension and compression can also be caused by the absence of bladder catheterization during labor, the presence of a bladder diverticulum and prolonged second stage of labour especially in association with foetal macrosomia [2]. However, neither foetal macrosomia nor high parity alone are predisposing factors for SRB [6]. Few cases of SRB after urethral catheterization without other predisposing factors have been described in the literature [7]. To prevent this kind of complication, it is recommended to use catheters of not too thin a gauge and avoid deep introduction of the catheter into the bladder in the final phase of labor [2]. In our case, the predisposing factor for SRB was forceps delivery on the one hand and the abdominal compression maneuvers (application of fundal pressure) on the other hand especially that the rupture was located at the dome which is the thinnest area of the bladder [6]. The real role of compression maneuver is understudied [2].

Concerning the reported clinical manifestations of SRB, the most frequently described symptoms in order of frequency are: abdominal pain, abdominal distension, fever, oliguria, hematuria and vomiting [2]. Biologically, acute renal failure sequel to systemic absorption of urea and creatine has been reported by some authors [2]. The first-line radiological examinations usually performed in this context are: trans-abdominal ultrasound and abdomino-pelvic CT scan. However, their contribution remains limited in the diagnosis of SRB, such as in our case: in both abdominal ultrasound and CT scan, the bladder seemed healthy. The uroscanner remains the gold standard if SRB is suspected [8]. The definitive diagnosis remains surgical. In fact, the presence of a postpartum effusion can have several other causes such as hepatic arterial thrombosis, acute pancreatitis, intestinal perforation [9], rupture or torsion of an ovarian cyst [10].

The treatment of SRB is surgical repair and urinary rest [8]. Surgical treatment consists of removing urine from the peritoneal cavity, closing the rupture and establishing a good bladder emptying [4]. Laparotomy is the standard surgical approach in this condition. Only one case of laparoscopic surgical treatment has been recently reported [2]. A bladder catheter should be kept for a few days postoperatively. In our case, we opted for a laparoscopic exploration and a conversion to laparotomy was performed due to technical difficulties (adhesions). The SRB was objectified and therefore sutured. The bladder catheter was kept for ten days.

## Conclusion

Despite being a rare condition, post-partum SBR should be considered in cases of abdominal pain, effusion and features of renal failure following delivery. It is associated with high maternal mortality owing majorly to late diagnosis. Treatment should be prompt and appropriate.
